# Influence of Agronomic Practices on the Antioxidant Compounds of Pigmented Wheat (*Triticum aestivum* spp. *aestivum* L.) and Tritordeum (× *Tritordeum martinii* A. Pujadas, nothosp. nov.) Genotypes

**DOI:** 10.1021/acs.jafc.3c02592

**Published:** 2023-08-29

**Authors:** Claudia Sardella, Barbora Burešová, Zora Kotíková, Petr Martinek, Raffaele Meloni, Luboš Paznocht, Francesca Vanara, Massimo Blandino

**Affiliations:** † Department of Agricultural, Forest and Food Sciences, University of Turin, Largo Paolo Braccini 2, Grugliasco, 10095 Turin, Italy; ‡ Department of Chemistry, Faculty of Agrobiology, Food and Natural Resources, Czech University of Life Sciences Prague, Kamýcká 129, Suchdol, 16500 Prague, Czech Republic; § Agrotest Fyto, Ltd., Havlíčkova 2787/121, 76701 Kroměříž, Czech Republic

**Keywords:** *Triticum aestivum*, × *Tritordeum martinii*, pigmented cereals, nitrogen, phenolic acids, anthocyanins, carotenoids, antioxidant capacity, technological quality

## Abstract

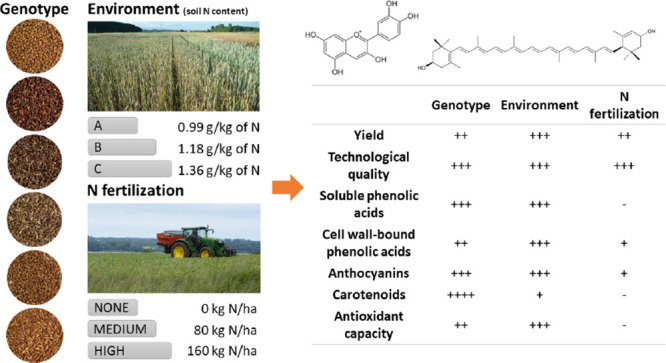

Twelve pigmented wheat genotypes, one tritordeum, and one common wheat were grown in three field experiments under varying nitrogen (N) fertilization rates to investigate the contributions of genotype, environment, and fertilization on the levels of phenolic acids, anthocyanins, carotenoids and antioxidant capacity of the grains. Soluble phenolic acids increased significantly (+16%) in the environment with high soil N content, while bound phenolic acids and anthocyanins decreased (−16 and −57%). N fertilization affected the agronomic and qualitative traits but had limited effects on some bioactive compounds (bound phenolic acids and anthocyanins). The greatest differences appeared among the color groups and within the same color types, with the black group showing the most anthocyanins and phenolic acids (34.4 and 1207 mg·kg^–1^) and the highest antioxidant capacity. Some of the cultivars could be promising for the development of innovative supply chains and the production of functional foods, as they showed good yield and quality performances, and good antioxidant features.

## Introduction

Bread wheat (*Triticum aestivum* spp. *aestivum* L.) products are the basis of most human diets across the globe, and they contribute to a great extent to the daily intake of antioxidant compounds and the prevention of aging processes caused by oxidative stress. Indeed, the consumption of whole-meal wheat has been associated with multiple health benefits, which are related to the high dietary fiber content and to the synergetic action of several bioactive compounds, such as micronutrients and phytochemicals.^1^ The most consumed wheat types are the white- and red-grained ones.^2^ However, some newly developed pigmented wheat varieties have been receiving a great deal of attention from breeders and scientists around the world as they appear to be a valuable source of protective phytochemicals, particularly those responsible for conferring the color to the kernels.^3^ Anthocyanins and carotenoids, apart from giving attractive blue-purple and yellow-orange colors to grains, respectively, have been associated with numerous biological activities^4^ and are regarded as potent antioxidant compounds that can add functionality to the energy-rich cereal matrix. Pigmented wheat exists in different forms, which depend on the type and location of the pigments within the kernel layers. Other works have confirmed that purple wheat contains anthocyanins in the pericarp layer (purple pericarp - Pp) and blue wheat in the aleurone layer (blue aleurone - Ba),^3^ whereas black wheat accumulates anthocyanins in both layers, combining the genetics of both purple and blue wheat (Pp + Ba).^5^ On the other hand, wheat genotypes containing high concentrations of carotenoids in the starchy endosperm are characterized by a bright yellow color of the kernels (yellow endosperm - Ye).^3^ Apart from innovative Ye wheat varieties, the novel cereal species tritordeum (× *Tritordeum martinii* A. Pujadas, nothosp. nov.) has shown promise, because of its high carotenoid content in the grain, which has been found to be more than five times higher than that of durum wheat.^6^ Furthermore, some tritordeum cultivars exhibit similar technological properties to those of bread wheat and therefore show a good bread-making aptitude.^7^ Thus, these novel and unconventional genotypes might be valuable resources to increase the nutritional value of wheat-derived food products. Nevertheless, the main challenge for their exploitation on a large scale is their lower yield than commonly cultivated non-pigmented varieties.^2,7^ However, different agricultural practices can be adopted to grow wheat and tritordeum with the aim of improving both the grain yield and quality. Since these techniques can have an effect on the concentration of health-promoting substances,^8−11^ more knowledge on the impact of different agronomic conditions on the phytochemical concentrations of these grains is needed to understand whether the agricultural management practices usually applied to common wheat could be helpful in enhancing the yield and agronomic performances of innovative pigmented genotypes, without having any detrimental effect on their high nutritional value. Nitrogen (N) is a fundamental nutrient for plant growth, and the supply of N is a relevant agronomic practice for crop production, as it influences the yield, the protein content, and the technological quality of the grains.^12^ The effect of N fertilization on the phytochemical concentrations of grains has been reported for different cereal crops.^13−15^ However, few data are available on pigmented cereals, and, at present, there is little information about the effect of the N fertility of the soil on the phytochemical accumulation of the grains of wheat and tritordeum genotypes. Therefore, the aim of the present study has been to evaluate the relative contribution of the genotype, the environment (defined as a combination of location and year), and the N fertilization to the variation in the levels of bioactive compounds, such as soluble and bound phenolic acids, anthocyanins, and carotenoids, and in the antioxidant capacity of a selected set of non-traditional genotypes with pigmented grains, including anthocyanin- and carotenoid-rich registered varieties and breeding lines, and considering one known commercial variety as a control.

## Materials and Methods

### Experimental Design

**Figure 1:**
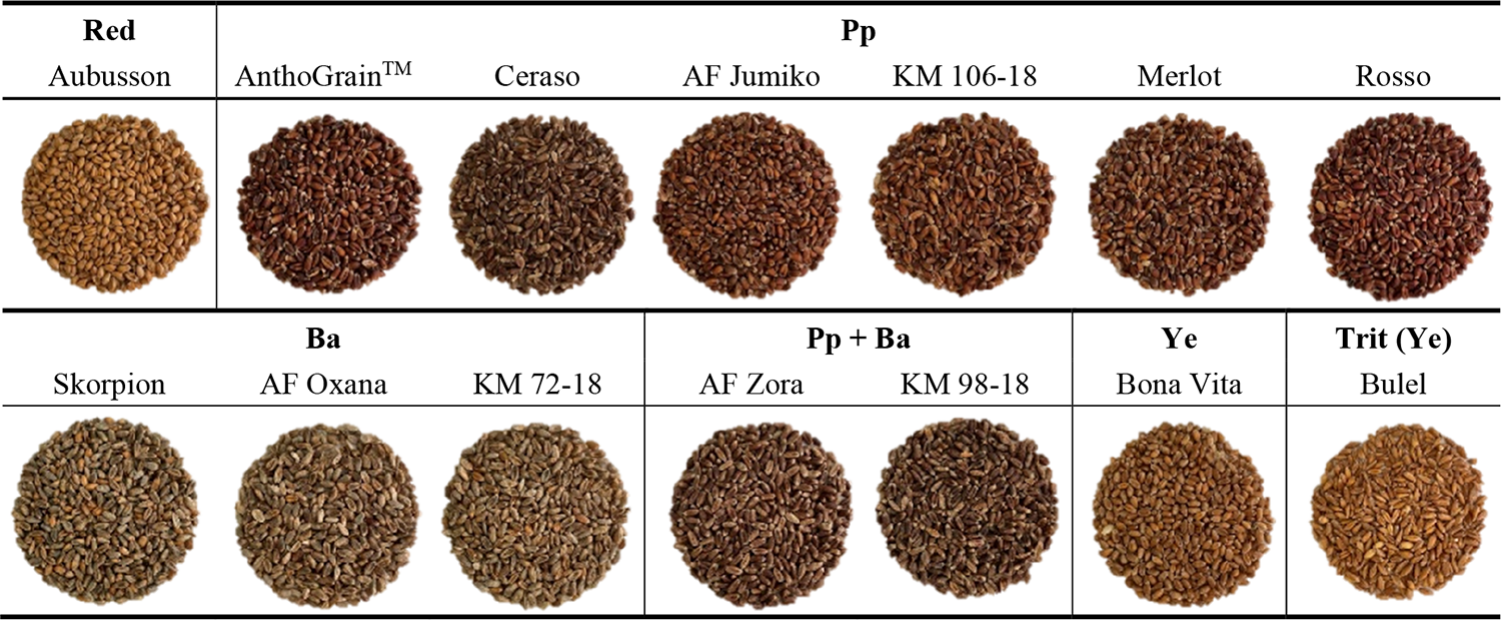
Wheat and tritordeum genotypes grouped according to the grain color, which depends on the type and position of the pigments within the kernel layers. Pp, purple pericarp; Ba, blue aleurone; Pp + Ba, black grain; Ye, yellow endosperm; Trit, tritordeum.

Thirteen bread wheat genotypes and one tritordeum genotype were used in this study. Among the considered wheat genotypes, there were six with Pp, three with Ba, two with Pp and Ba, one with Ye, and one common (red) bread wheat, which was chosen as a widely cultivated variety and used as a control (Figures 1 and S1; Table 1). The studied genotypes were grown during the 2018–19 growing season in two different locations in north-west Italy (Cigliano, Piedmont, 45° 18′ N, 8° 01′ E; Carmagnola, Piedmont, 44° 50′ N, 7° 40′ E). The field trial was repeated during the 2019–20 growing season in Cigliano. The field in Cigliano was characterized by a shallow sandy-loam soil (Typic Hapludalfs), whereas the one in Carmagnola was characterized by a deep silty-loam soil (Typic Udifluvents). The N content of the soil in both locations was tested at the end of the winter period (Table 2). The soils were sampled at a depth of 0–30 cm, using Eijkelkamp cylindrical augers, at the beginning of March, just before the N fertilization at the tillering growth stage (GS 23, according to Zadoks et al.).^16^ The daily precipitations and temperatures were measured at meteorological stations located near the two experimental areas (Table 3). Due to the remarkably different growing conditions observed in the field trials, both in terms of soil characteristics, mainly as N content, and weather conditions, three different environments were defined as a combination of location and year and were set up as follows: A (Cigliano, 2019–20 growing season), B (Cigliano, 2018–19), and C (Carmagnola, 2018–19). The genotypes were cultivated under N-input regimes of 0 kg of N·ha^–1^ (unfertilized), 80 kg of N·ha^–1^ (medium N fertilization), and 160 kg of N·ha^–1^ (high N fertilization). The total amount of administered N was split into two dosages to avoid leaching and to improve N availability, according to the ordinary N fertilization practices in the growing areas. The N fertilizer was provided manually and was evenly distributed as ammonium nitrate (granular, N 27%) at the tillering stage (GS 23) and at the beginning of stem elongation (GS 32).

**Table 1:** Wheat and Tritordeum Genotypes Compared in the Study and Their Origin^a^

type	genotype	in charge of breeding	year of release
red wheat (control)	Aubusson	Limagrain Italia S.p.A., Fidenza (PR), Italy	2002
Pp wheat	AnthoGrain^TM^	Hetland Seeds Ltd., Naicam, SK, Canada	2013
	Ceraso	Saatzucht Donau GesmbH. & CoKG, Austria	2014
	AF Jumiko	Agrotest Fyto, Ltd., Kroměříž, Czech Republic	2018
	KM 106-18	Agrotest Fyto, Ltd., Kroměříž, Czech Republic	breeding line
	Merlot	Saatzucht Donau GesmbH. & CoKG, Austria	2015
	Rosso	Saatzucht Donau GesmbH. & CoKG, Austria	2010
Ba wheat	Skorpion	Agrotest Fyto, Ltd., Kroměříž, Czech Republic	2011 (2012 - European Catalogue of Varieties)
	AF Oxana	Agrotest Fyto, Ltd., Kroměříž, Czech Republic	2019
	KM 72-18	Agrotest Fyto, Ltd., Kroměříž, Czech Republic	breeding line
Pp + Ba wheat	AF Zora	Agrotest Fyto, Ltd., Kroměříž, Czech Republic	2021
	KM 98-18	Agrotest Fyto, Ltd., Kroměříž, Czech Republic	breeding line
Ye wheat	Bona Vita	Istropol Solary a.s., Horné Mýto, Slovakia	2011
tritordeum (Ye)	Bulel	Arcadia S.p.A., Pamplona, Spain	2015

a Pp, purple pericarp; Ba, blue aleurone; Pp + Ba, black grain; Ye, yellow endosperm.

**Table 2:** Main Information on the Trial and on the Physical and Chemical Characteristics of the Soil for the Field Experiments Conducted in the 2018–20 Period in North-West Italy^a^

		environments		
parameters	measuring unit	A	B	C
growing season		2019–20	2018–19	2018–19
location		Cigliano (VC)	Cigliano (VC)	Carmagnola (TO)
geographic coordinates		45° 18′ N, 8° 01′ E	45° 18′ N, 8° 01′ E	44° 50′ N, 7° 40′ E
altitude	m	236	236	245
soil (USDA classification)		Typic Hapludalfs	Typic Hapludalfs	Typic Udifluvents
sand (2–0.05 mm)	%	41.7	39.4	32.1
silt (0.05–0.002 mm)	%	47.5	52.3	65.5
clay (<0.002 mm)	%	10.8	8.3	5.3
pH		5.9	6.6	8.1
organic matter	%	1.51	1.59	2.01
C/N		9.4	8.8	8.6
cation exchange capacity (C.E.C.)	Cmol(+)·kg^–1^	10.2	10.6	9.8
exchangeable potassium	mg·kg^–1^	69	135	72
available phosphorus	mg·kg^–1^	42	25	15
total N	g·kg^–1^	0.99	1.18	1.36
estimated mineralized N^b^	kg·ha^–1^·year^–1^	134	159	201
AUCGC with different N rates (kg of N·ha^–1^)				
0	NDVI-Day	38.6	57.1	68.4
80	NDVI-Day	49.0	61.6	68.4
160	NDVI-Day	57.0	66.5	70.2
GY with different N rates (kg of N·ha^–1^)				
0	t·ha^–1^	3.1	3.9	6.5
80	t·ha^–1^	4.9	4.7	6.7
160	t·ha^–1^	5.8	5.8	6.4
GPC with different N rates (kg of N·ha^–1^)				
0	%	10.9	10.6	12.9
80	%	10.7	10.9	13.8
160	%	11.5	12.5	14.6

a The data reported for the area under the canopy greenness curve (AUCGC), grain yield (GY), and grain protein content (GPC) refer to the averages of all the compared genotypes and replications.

b Calculated on the basis of total N and the physical parameters of the soil, according to Grignani et al.^25^

**Table 3:** Monthly Rainfall, Rainy Days, and Growing Degree Days (GDDs)^a^ from Sowing (November) to the End of the Ripening Stage (June) in the Field Experiments

environments	months	rainfall (mm)	rainy days (n°)	GDDs (Σ °C·d^–1^)
A	November	314	12	249
Cigliano	December	132	8	193
2019–20	January	5	1	168
	February	1	0	229
	March	62	7	285
	April	81	10	414
	May	122	7	579
	June	113	7	624
	November–June	830	52	2741
	November–March	514	28	1124
	April–June	316	24	1617
B	November	124	5	292
Cigliano	December	11	1	151
2018–19	January	6	1	141
	February	43	7	195
	March	17	4	314
	April	116	7	393
	May	178	9	478
	June	40	4	667
	November–June	535	38	2632
	November–March	200	18	1093
	April–June	335	20	1539
C	November	118	17	281
Carmagnola	December	9	3	110
2018–19	January	7	2	62
	February	32	3	133
	March	4	1	305
	April	110	12	377
	May	97	12	484
	June	36	7	694
	November–June	413	57	2445
	November–March	170	26	890
	April–June	243	31	1555

a Accumulated growing degree days for each month using a 0 °C base. Source: Agro-meteorological service of the Piedmont region.

Thus, the treatments compared at each location were factorial combinations of 14 genotypes (G), 3 environments (E), and 3 N fertilization rates (N). The treatments were assigned to experimental units using a completely randomized block design, with a 10.5 m^2^ plot (7 × 1.5 m) and three replications (*n* = 378). The agronomic techniques commonly adopted in the area were applied to all plots. Briefly, the previous crop in all the experiments was maize, the field was plowed each year, incorporating the debris into the soil, and this was followed by disk harrowing to prepare a suitable seedbed. Planting was conducted in 12 cm wide rows at a seeding rate of 450 seeds·m^–2^ in October or November (Table S1). The weed control was conducted with mesosulfuron-methyl and iodosulfuron-methyl-sodium at stem elongation (GS 31). A fungicide (prothioconazole + tebuconazole) and an insecticide (deltamethrin) were applied to all the plots at anthesis (GS 65) to minimize the negative impact of fungal diseases and insects. Harvesting was carried out using a Walter Wintersteiger cereal plot combine harvester.

### Grain Yield and Physical Parameters

The area under the canopy greenness curve (AUCGC) was calculated from normalized difference vegetation index (NDVI) measurements taken throughout the growing seasons, according to De Santis et al.^17^ The grain yield (GY) was calculated on a plot basis and adjusted to a 13% moisture content. The harvested grains were mixed thoroughly, and 2 kg grain samples were taken from each plot to determine the grain moisture content and the test weight (TW), which was done by means of a GAC 2000 Grain Analyzer (Dickey-John Corp., Auburn, IL, USA). The thousand-kernel weight (TKW) was determined on two 200 kernel sets of each sample, using an electronic balance.

### Qualitative Parameters

Representative subsamples (500 g) were ground to whole-meal using a laboratory centrifugal mill equipped with a 1 mm sieve (Model ZM-200, Retsch, Haan, Germany). The grain protein content (GPC) was determined according to the AACC method 39-10.01^18^ by means of an NIR System Model 6500 (FOSS-NIRSystems, Laurel, MD, USA), as was the ash content. All the samples were ground to a fine powder (particle size <500 μm) with a Cyclotec 1093 sample mill (Foss, Padua, Italy) and stored at −25 °C, prior to the chemical analyses. The moisture content, which was determined to express the concentrations of phenolic acids, anthocyanins, and carotenoids and the antioxidant capacity results on a dry weight (DW) basis, was obtained by oven-drying the <500 μm samples at 105 °C to a constant weight.

### Chemical Analyses

#### Chemicals

2,2′-Azino-bis(3-ethylbeenzothiazoline-6-sulfonic acid) diammonium salt (ABTS), ethanol (CHROMASOLV, 99.8%), ethyl acetate (CHROMASOLV, 99.8%), (±)-6-hydroxy-2,5,7,8-tetramethylchromane-2-carboxylic acid (Trolox, 97%), hydrochloric acid (HCl, 37%), iron(III) chloride hexahydrate (FeCl_3_·6H_2_O, ≥98%), methanol (CHROMASOLV, 99.9%), potassium sulfate (≥99%), sodium hydroxide (≥98%), 2,4,6-tris(2-pyridyl)-*s*-triazine (TPTZ), butylated hydroxytoluene (BHT, ≥99%), *tert*-butyl methyl ether (HPLC grade), and phenolic acid standards (caffeic acid ≥98%, *p*-coumaric acid ≥98%, ferulic acid ≥99%, gallic acid ≥99%, protocatechuic acid ≥99%, *p*-hydroxybenzoic acid ≥99%, sinapic acid ≥98%, syringic acid ≥95%, and vanillic acid ≥97%) were purchased from Sigma-Aldrich (St. Louis, MO, USA). Anthocyanin standards (cyd-3-glu, cyanidin-3-glucoside; cyd-3-rut, cyanidin-3-rutinoside; dpd-3-glu, delphinidin-3-glucoside; dpd-3-rut, delphinidin-3-rutinoside; pnd-3-glu, peonidin-3-glucoside), all with a purity ≥97%, were purchased from Polyphenols (Sandnes, Norway). Lutein (≥95%) and zeaxanthin (≥98%) standards were obtained from Extrasynthese (Genay, France). Methanol, ethanol, acetone, and hexane, all p.a. grade, were obtained from Lachner (Neratovice, Czech Republic). Formic acid (99–100%) was purchased from VWR (Radnor, PA, USA). Acetonitrile (HPLC grade) was purchased from Carlo Erba (Milan, Italy). Water (HPLC grade) was obtained from an ELGA PURELAB Ultra system (M-medical, Cornaredo, Milan, Italy).

#### Extraction and Quantification of the Phenolic Acids, Carotenoids, and Anthocyanins

The soluble phenolic acids (SPAs) and cell wall-bound phenolic acids (CWBPAs) were individually extracted by a two-step method previously described.^19^ A common extraction step was performed using an ethanol–water solution (80:20, *v*/*v*), then the SPAs were determined after alkaline hydrolysis of the ethanol:water extract, whereas CWBPAs were determined after alkaline hydrolysis of the solid sample residue. After acidification of the hydrolysates and liquid–liquid extraction with ethyl acetate, the organic phase was evaporated to dryness under a nitrogen stream. The dry residue was reconstituted with 80:20 (*v*/*v*) methanol:water solution, filtered, and analyzed by means of high-performance liquid chromatography with diode array detection (HPLC/DAD). The detailed sample preparation procedure as well as the chromatographic conditions are described in Giordano et al.^19^ Phenolic acids were identified using the retention times and the UV/vis spectra of their respective standards. Solutions of individual phenolic acid standards were prepared and diluted to different concentrations to obtain calibration curves for quantification purposes.

The carotenoids were extracted with a mixture of ethanol:acetone:hexane (1:1:2, *v*/*v*/*v*). After evaporation of the extract under vacuum at 40 °C, the dry residue was reconstituted with ethanol:acetone (3:2, *v*/*v*) containing 0.2% BHT, filtered, and analyzed by means of HPLC/DAD. The detailed method of carotenoid analysis is described in Paznocht et al.^20^ Quantification was carried out by means of external calibration in the range of 0.1–10 μg·ml^–1^.

The anthocyanins were extracted with a mixture of methanol and 1 M HCl (85:15, *v*/*v*) as reported by Ficco et al.^21^ The extract was then centrifuged at 8228 rcf for 10 min (5810R, Eppendorf, Hamburg, Germany) after freezing at −18 °C and evaporated under vacuum at 45 °C (Rotavapor R-200, Büchi Labortechnik, Flawil, Switzerland). The dry residue was reconstituted with 2 mL of the extraction mixture, filtered through a PTFE microfilter (0.22 μm), and analyzed by means of HPLC/DAD (Ultimate 3000, Thermo Fisher Scientific, Waltham, MA, USA). The analytes were separated by gradient elution on a Gemini analytical column (C18, 150 × 4.6 mm, *S* = 3 μm) and a guard column C18 (4 × 3 mm), both from Phenomenex (Torrance, CA, USA). Mobile phase A consisted of formic acid:H_2_O (1:99, *v*/*v*), and mobile phase B consisted of 10% formic acid:H_2_O (1:99, *v*/*v*), 50% methanol, and 40% acetonitrile. The gradient was expressed in terms of mobile phase B: 0–1 min 10% B, 1–5 min 20% B, 5–20 min 50% B, and 20–23 min 95% B, and this was followed by column flushing and equilibration over the next 14 min. The operating conditions were as follows: flow rate at 0.5 mL·min^–1^, column temperature at 40 °C, autosampler temperature at 15 °C, injection volume at 5 μL, time of analysis at 37 min, detection wavelength at 525 nm, and spectral acquisition at 300–600 nm. Identification was performed by comparing the retention times and absorption spectra with those of analytical standards. Quantification was carried out by means of external calibration, which was based on the peak area (0.05–10 μg·mL^–1^). The exact concentrations of the analytical standards were calculated using molar absorptivity (ε).^22^

The results of all the determined and quantified phytochemicals by means of HPLC separations (SPAs, CWBPAs, carotenoids, and anthocyanins) were expressed as mg·kg^–1^ of DW.

#### Determination of the Antioxidant Capacity by Means of ABTS (AC_ABTS_) and FRAP Assays (AC_FRAP_)

ABTS and ferric reducing antioxidant power (FRAP) assays were employed to determine the AC of the flour following the QUENCHER procedure (direct measurement on solid samples), as described by Serpen et al.^23^ Both of the methods allow the evaluation of the electron-donating potency of the antioxidant compounds of the solid matrix, by scavenging the synthetic ABTS radical cation and by reducing the Fe^III^ to Fe^II^.

For AC_ABTS_ analysis, the whole-meal flour was mixed with the ABTS reagent, which was previously prepared by reacting an aqueous solution of ABTS with potassium persulfate and ethanol in a 1:1 *v*/*v* ratio. The mixture was allowed to react on a shaker at 20 °C for 30 min, immediately followed by centrifugation and measurement of absorbance at 734 nm. For the determination of AC_FRAP_, the whole-meal flour was mixed with FRAP working solution (10 mM TPTZ, 20 mM FeCl_3_, and 300 mM sodium acetate buffer of pH 3.6, 1:1:10, *v*/*v*/*v*) and methanol in a 99:1 *v*/*v* ratio. The mixture was allowed to react on a shaker at 20 °C for 120 min, immediately followed by centrifugation and measurement of absorbance at 593 nm. The detailed procedures for both assays are described in Serpen et al.^24^ The results were expressed as mmol Trolox equivalents (TE)·kg^–1^ of DW by means of a Trolox dose–response curve.

#### Statistical Analyses

The obtained data were compared, by means of an analysis of variance (ANOVA), to evaluate the effect of the genotype (G), the environment (E), the N fertilization (N), and their interactions on the yield, the grain qualitative parameters, and on the content of bioactive compounds of the whole-meal flour. Means were identified as significantly different (*p* < 0.05) on the basis of the Ryan–Einot–Gabriel–Welsch F (REGW-F) statistical test. Prior to the analysis, data were tested for normality and homoscedasticity. The analyses were carried out by means of the SPSS for Windows statistical package, version 28.0 (SPSS, Inc., Chicago, IL, USA). A principal component analysis (PCA) was performed to investigate the relationships between the bioactive compounds and the antioxidant properties of the analyzed genotypes grown under the three different environmental conditions. The data were first standardized by subtracting the means and dividing the result by the standard deviations of each variable. The multivariate analysis was carried out by means of the Past4Project for Windows data analyzer, version 4.03.

## Results and Discussion

### Meteorological Data and Agronomic Conditions

The meteorological data recorded during the two growing seasons in the three research sites are shown in Table 3. High rainfall occurred in the second growing season (2019–20), particularly in November and December, thus leading to a probable higher N leaching at the end of winter in environment A. Overall, the rainfall distributions and the temperatures from flowering (May) to the end of the ripening stage (June) were similar in all the considered production systems, thereby resulting in comparable weather conditions across the three environments. As far as the N content of the soil is concerned (Table 2), the environment C (Carmagnola, on a silty-loam deep soil) was higher than environments B and A (Cigliano, on a sandy-loam soil). According to soil physical and chemical parameters*,* the expected mineralized N within the growing season increased from environment A to B and C.^25^ The different plant N availability in the compared environments can be directly deduced from the crop response: the AUCGC index, which expresses both the plant vigor and the leaf chlorophyll content within the growing season and which is strictly influenced by the N availability,^17^ the GY, and the GPC increased proportionally from environment A to C (Table 2).

### Grain Yield and the Physical and Qualitative Parameters

**Figure 2:**
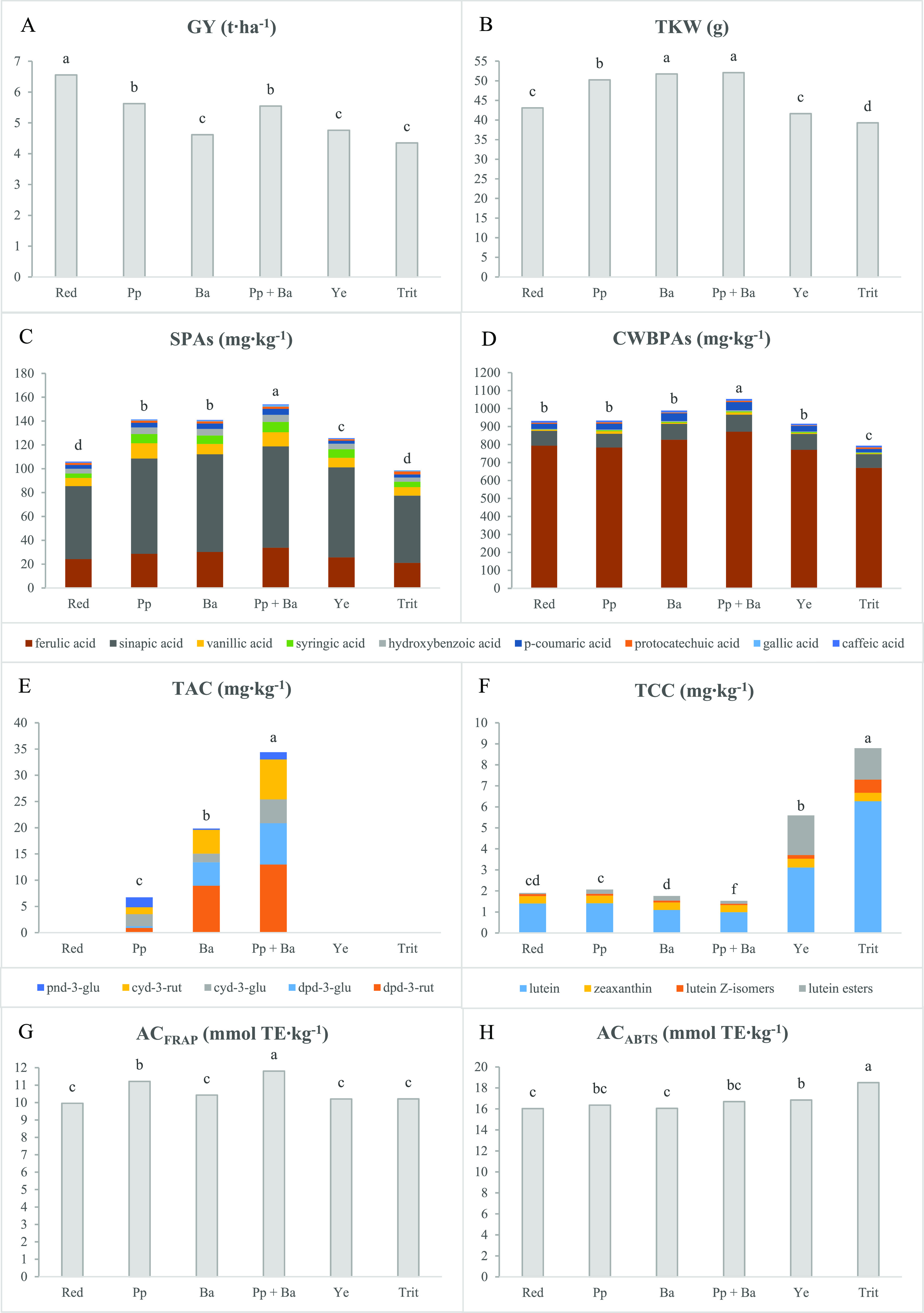
Comparison of the (A, B) agronomic traits, (C–F) bioactive compounds, and (G, H) antioxidant capacity of the investigated wheat and tritordeum genotypes grouped according to the grain color. Pp, purple pericarp; Ba, blue aleurone; Pp + Ba, black grain; Ye, yellow endosperm; Trit, tritordeum; GY, grain yield; TKW, thousand kernel weight; SPAs, soluble phenolic acids; CWBPAs, cell wall-bound phenolic acids; TAC, total anthocyanin content; pnd-3-glu, peonidin-3-glucoside; cyd-3-rut, cyanidin-3-rutinoside; cyd-3-glu, cyanidin-3-glucoside; dpd-3-glu, delphinidin-3-glucoside; dpd-3-rut, delphinidin-3-rutinoside; TCC, total carotenoid content; AC, antioxidant capacity (FRAP and ABTS assays). The results are expressed on a DW basis. Different letters above the columns indicate significant differences between the six groups at *p* (F) < 0.05, according to the REGW-F test.

Three-way ANOVA showed significant effects of all the three investigated factors (genotype, environment, and N fertilization) on the GY and TKW of the 14 evaluated cereal genotypes (Table 4). The factor that had the most important effect on GY was the environment (around 54% of the variation), followed by the N fertilization and the genotype (29 and 6%). On the other hand, the genotype was the factor that had the most effect on TKW, accounting for almost 70% of the variation. As expected, the recently developed pigmented genotypes showed a significantly lower productivity than the common red variety (Table 5), which had been chosen as it is one of the most cultivated varieties in North Italy, and it is characterized by a high-yield potential. However, a great variation was observed for GY and TKW among and within the color lines. Compared to the control, the lowest GY (−34 and −27%) and the lowest TKW (−9 and −3%) were observed for the Ye cereals, that is, tritordeum and wheat, respectively (Figure 2A,B). Similarly, a low average GY was recorded for the Ba wheats, whereas the Pp and Pp + Ba groups showed higher GY values for the pigmented genotypes, although they were still significantly lower than the control. The TKW trait showed differences among the groups, with Pp + Ba > Ba > Pp > red > Ye wheat > Ye tritordeum, possibly due to differences in their grain shapes as a result of the strong genotypic component.

**Table 4:** Level of Significance of the Three-Way ANOVA Analyses Performed to Evaluate the Contribution of the Genotype, the Environment, the N Fertilization, and Their Interaction on the Agronomic Traits, Bioactive Compounds, and Antioxidant Capacity of the Whole-Meal Flour of the Investigated Wheat and Tritordeum Genotypes^a^

factor	GY		TKW		SPAs		CWBPAs		TAC		TCC		AC_ABTS_		AC_FRAP_	
genotype (G)	6.4	***	69.9	***	44.0	***	13.3	***	45.5	***	97.3	***	1.6	***	29.1	***
environment (E)	54.0	***	21.8	***	41.1	***	57.9	***	49.4	***	1.2	***	97.3	***	47.2	***
N fertilization (N)	29.1	***	0.9	*	0.6		10.8	***	0.6	***	0.1	*	0.1		3.4	
G × E	0.9	***	4.8	***	8.9	***	10.7	***	4.1	***	1.0	***	0.3	***	12.1	***
G × N	0.1		0.7	***	2.3	***	0.9		0.1		0.1	**	0.2	***	3.1	**
E × N	9.2	***	1.2	**	0.1		4.5	***	0.1		0.1	**	0.3	**	0.6	
G × E × N	0.1		0.4	*	2.0	***	1.2	**	0.1		0.1		0.1	***	2.9	**
error	0.1		0.3		0.9		0.7		0.1		0.0		0.1		1.6	

a The results are expressed as percentages of the total mean square. GY, grain yield; TKW, thousand kernel weight; SPAs, soluble phenolic acids; CWBPAs, cell wall-bound phenolic acids; TAC, total anthocyanin content; TCC, total carotenoid content; AC, antioxidant capacity (ABTS and FRAP assays). The marked factors are statistically significant, according to the REGW-F test [(*) *p* (F) < 0.05, (**) *p* (F) < 0.01, and (***) *p* (F) < 0.001].

The GY and TKW were influenced by the environment to a great extent, although at different rates in relation to the genotype (Table 4). A significantly higher average yield was observed in the environment with high soil N content (C, 6.5 t·ha^–1^), followed by that of the B environment (4.8 t·ha^–1^), both significantly different (+42 and +4%) from A (4.6 t·ha^–1^), as shown in Table 5. In agreement with previous studies,^12,13,17,26^ the N fertilization rate affected the productivity of all the tested varieties to a great extent, but it did so at a different rate in relation to the genotype (Table 4). The application of 160 and 80 kg of N·ha^–1^ resulted in increases in GY of about 34 and 21%, respectively, when compared to 0 kg of N·ha^–1^ (6.0 and 5.4 vs 4.5 t·ha^–1^, Table 5). As expected, TKW dropped along with the N fertilization rate and the soil N content in the environments as a trade-off between the seed number and the seed size.

**Table 5:** Effect of the Genotype, the Environment, and the N Fertilization on the Agronomic Traits, Bioactive Compounds, and Antioxidant Capacity of the Whole-Meal Flour of the Investigated Wheat and Tritordeum Genotypes^a^

factor	source of variation		GY (t·ha^–1^)		TKW (g)		SPAs (mg·kg^–1^)		CWBPAs (mg·kg^–1^)		TAC (mg·kg^–1^)		TCC (mg·kg^–1^)		AC_ABTS_ (mmol TE·kg^–1^)		AC_FRAP_ (mmol TE·kg^–1^)	
genotype	Red	Aubusson	6.6	a	43.1	f	106	gh	931	defg	<LOD		1.9	f	16.0	cd	10.0	c
	Pp	AnthoGrain^TM^	4.8	e	51.9	b	142	cde	967	cdef	13.9	e	2.7	c	17.4	b	12.2	a
		Ceraso	5.8	bc	50.1	cd	146	bcd	992	bcde	14.9	e	1.7	g	17.0	bc	11.8	ab
		AF Jumiko	6.1	b	45.2	e	133	ef	831	hi	1.2	g	1.7	g	16.6	bc	11.3	b
		KM 106–18	5.4	cd	53.2	a	145	bcd	926	defg	1.9	g	2.1	e	16.7	bc	10.4	c
		Merlot	6.0	b	51.2	bc	137	def	891	fgh	2.5	g	1.6	g	15.6	de	10.2	c
		Rosso	5.7	bcd	49.9	d	146	bcd	995	abcd	6.4	f	2.6	d	15.0	e	11.5	ab
	Ba	Skorpion	3.6	g	51.9	b	111	g	1051	abc	18.9	d	1.9	f	15.5	de	10.3	c
		AF Oxana	5.5	cd	49.8	d	147	bcd	842	ghi	18.8	d	1.7	g	17.1	b	10.6	c
		KM 72-18	4.8	e	53.5	a	165	a	1073	a	21.9	c	1.7	g	15.6	de	10.5	c
	Pp + Ba	AF Zora	5.3	d	50.9	bcd	153	bc	1041	abc	42.9	a	1.7	g	16.7	bc	11.9	ab
		KM 98-18	5.8	bcd	53.3	a	155	ab	1066	ab	25.9	b	1.3	h	16.7	bc	11.7	ab
	Ye	Bona Vita	4.8	f	41.6	g	126	f	916	efg	<LOD		5.6	b	16.8	bc	10.2	c
	Trit	Bulel	4.3	f	39.3	h	99	h	793	i	<LOD		8.8	a	18.5	a	10.2	c
environment		A	4.6	c	49.5	a	126	c	1022	a	22.9	a	2.5	c	12.5	c	11.3	a
		B	4.8	b	49.6	a	138	b	968	b	13.4	b	2.8	a	17.7	b	10.5	c
		C	6.5	a	47.5	b	145	a	859	c	9.8	c	2.6	b	19.5	a	10.9	b
N fertilization (kg of N·ha^–1^)		0	4.5	c	48.9	ab	136	a	993	a	16.0	a	2.7	ab	16.5	a	11.0	a
		80	5.4	b	49.0	a	137	a	927	b	15.5	a	2.7	a	16.4	a	10.9	a
		160	6.0	a	48.6	b	135	a	933	b	14.6	b	2.6	b	16.6	a	10.8	a

a Pp, purple pericarp; Ba, blue aleurone; Pp + Ba, black grain; Ye, yellow endosperm; Trit, tritordeum; GY, grain yield; TKW, thousand kernel weight; SPAs, soluble phenolic acids; CWBPAs, cell wall-bound phenolic acids; TAC, total anthocyanin content; TCC, total carotenoid content; AC, antioxidant capacity (ABTS and FRAP assays); LOD, limit of detection. The results are expressed on a DW basis. Means followed by different letters are significantly different, according to the REGW-F test. The ANOVA level of significance is shown in Table 4.

The E × N was the interaction that influenced GY the most (around 9% of the variation), whereas it contributed to TKW to a lesser extent (1%), although the effect was still significant (Table 4). The increase in GY, as a consequence of N fertilization, was not significant in the C environment, presumably because of the higher N content of the soil in the site (data not shown).

Table S2 shows the qualitative parameters of the whole-meal flour of the investigated genotypes. The wheat group with Ba was characterized by the lowest mean TW (73.8 kg·hL^–1^, −5% with respect to the control). The pigmented genotypes showed similar or higher GPC (11.5, 12.3, 12.5, and 12.9% for the Pp, Ba, Pp + Ba, and Ye groups, respectively) than the commercial variety (11.5%), as previously observed by some other authors,^21,27^ and were thus characterized by good product-based utilization features. A comparison of the technological and rheological properties of the wheat samples is provided in Table S3. The farinograph evaluation and the baking test showed a comparable bread-making aptitude of the pigmented wheat genotypes to that of the control.

### Soluble and Cell Wall-Bound Phenolic Acids

Small cereals contain phenolic compounds as secondary metabolites that are naturally synthesized in response to various biotic and abiotic stress factors, and they are mainly appreciated for their antioxidant properties. Phenolic acids constitute one of the main groups of phenolic compounds that have been identified in wheat. They exist in cereals in soluble-free, soluble-conjugated, and insoluble-bound forms, with most of them being present in the aleurone layer and in the bran linked to cell wall materials, such as polysaccharides and lignans, through ester bonds.^28^ The insoluble fraction has been associated with more effective health improvement than the soluble forms. In fact, phenolics bound to dietary fiber may survive upper gastrointestinal digestion and be released through the activity of the intestinal microflora, thus exerting antioxidant effects in the colon or other tissues after absorption and slow release into the bloodstream.^29^ In this work, soluble-free and conjugated acids were extracted together and analyzed as free aglycones by HPLC, since the free form usually constitutes a small proportion of the total content (<1%).^28,30,31^ As expected, CWBPAs represented most of the total content of the phenolic acids, on average accounting for more than 87%. The HPLC/DAD analysis revealed that ferulic acid was the primary phenolic acid in the bound form (Figure 2D), representing more than 84% of the total CWBPAs in all the genotypes, followed by sinapic (9%) and *p*-coumaric acids (4%), whereas sinapic and ferulic acids constituted the majority of SPAs (57 and 21%), followed by a small share of vanillic acid (8%). The relative distribution of SPAs and CWBPAs did not differ to any great extent between genotypes or between color groups, although other works have described different phenolic compound profiles between wheat genotypes.^32^ However, Paznocht et al.^30^ observed that the share of individual phenolic acids over the total content was very similar across the analyzed pigmented wheat groups, and the shares of soluble and bound forms over the total concentration (8 and 92%) they observed were similar to those of the present study. These results are in line with the values reported by Martini et al.^31^ (14 and 86%), who did not observe a great variability in the SPA and CWBPA spectrum across durum wheat genotypes (with the exception of the soluble free forms, which, however, accounted for less than 1% of the total phenolic acids). In the same way as for wheat, the CWBPA content of tritordeum was higher than those of the SPAs. Moreover, the phenolic acid profile was similar to the one observed for the pigmented wheats, a result that is consistent with previously reported findings. Giordano et al.^19^ observed that the SPA and CWBPA profiles of tritordeum were closer to those of durum and bread wheat cultivars than to those of barley. Navas-Lopez et al.^33^ reported a similar percentage of ferulic acid in tritordeum and in wheat, with respect to the total phenolic compounds.

All the three factors analyzed with three-way ANOVA showed significant effects on the CWBPA content, whereas only the genotype and the environment had significant effects on the SPA content (Table 4). The genotype accounted for most of the variation observed for the SPAs (44%) and, albeit to a lesser extent, for the CWBPAs (13%). Overall, all the pigmented groups had similar or higher concentrations of SPAs and CWBPAs than the control, with the exception of tritordeum (Figure 2C,D). This result is in agreement with previous studies performed on the whole-meal flour of pigmented genotypes. Paznocht et al.^30^ classified the analyzed pigmented wheat groups according to the total phenolic acids in wholegrain flour in descending order as follows: Ba > Pp > Ye > red. Ma et al.^34^ reported a 20% higher phenolic acid content in Pp wheats than in control varieties. In the present study, the Pp + Ba genotypes had the highest contents of both SPAs and CWBPAs, that is, 45 and 13% more than the control, a result that is in agreement with previous works performed on whole-meal black wheat flour.^5,35^ As shown in Table 5, the highest concentrations of both SPAs and CWBPAs were observed in the KM 72-18 Ba genotype (165 and 1073 mg·kg^–1^), while tritordeum presented the lowest values (99 and 793 mg·kg^–1^).

The marked effect of the environment factor on the SPAs and CWBPAs (41 and 58%) resulted in higher concentrations of SPAs in the C (+14%) and B (+9%) environments than in the A one (Table 5). The CWBPA content showed an opposite trend, with increases of 19 and 13% in the grains obtained from the A and B environments, compared to the C one. The N fertilization rate induced consistent CWBPA results, with the highest values being measured in the samples cultivated in the unfertilized plots (+6% with respect to plots treated with 160 kg of N·ha^–1^), while no significant differences were detected on the SPA contents at different N rates.

The interaction that significantly influenced both the SPA and CWBPA contents the most was G × E (around 9 and 11% of the variation), followed by G × N for the SPAs (2%), with the N effect only being significant for the Ceraso and AnthoGrain^TM^ genotypes (Pp), and E × N for the CWBPAs (4%). Specifically, the higher CWBPA content associated with lower N fertilization rates was noticeable in B and C, whereas no significant difference was observed in the A environment (data not shown).

The effect of N on the contents of the phenolic compounds of plants in greenhouse or growth chamber experiments has been studied by various authors.^35−37^ As wheat phenolics seem to be more abundant in immature grains (in the milky and soft stages) and to decrease during the maturity process,^13,32^ such types of stress as N deficiency, which interferes with the normal maturation process, may lead these secondary metabolites to remain in higher concentrations in mature kernels. Furthermore, according to the “C/N balance theory”, when N availability is limited, plants change their metabolism toward carbon-containing compounds, such as starch, cellulose, and non-N-containing secondary metabolites, including phenolic acids and terpenoids. Moreover, the accumulation of phenolic acids has been associated with the expression of genes involved in the phenylpropanoid pathway.^36^ Under conditions of reduced N availability, the phenylalanine ammonia lyase enzyme catalyzes the deamination of phenylalanine, thereby releasing ammonia and cinnamic acid. The produced ammonia can be recycled to generate the amino acids required for the biosynthesis of proteins and to sustain plant growth,^37^ while cinnamic acid can be directed toward different biosynthetic pathways to form various phenylalanine-derived phenolics, such as phenolic acids or anthocyanins.^36,38^ However, the aforementioned studies monitored the effect of an N deficiency on the leaf tissues under controlled conditions, whereas the results from experiments performed under field conditions on cereal grains offered conflicting information. N fertilization has been reported to induce little^9^ or no change^8^ in the phenolic content of wheat grains, while other authors have observed an increase in the soluble,^13^ or even in both the soluble and insoluble,^15^ phenolic acid fractions. These contrasting results may be due to the different field conditions in the above-mentioned studies, such as in the meteorological trends, in the physical and chemical characteristics of the soil and/or in the agronomic management. In the present study, the environment, by comparing experiments with different soil N contents, had a more pronounced effect on the accumulation of phenolic acids than the N fertilization rate. A first explanation is related to the role that the different meteorological trends and the complex of soil properties could have on the accumulation of these bioactive compounds.^8,9,11,13−15^ Furthermore, according to the reported agronomic indexes (e.g. AUCGC, GY, and GPC), which are strictly related to the soil N availability for the plant, the environment was characterized by a more marked inter-experiment variation than that among N fertilization rates, thus suggesting a greater difference in crop N nutrition within the compared environments. However, little is known about the effect of the soil N content on the accumulation of secondary metabolites, which are assumed to have beneficial effects on human health, in pigmented wheat grains. Zrcková et al.^39^ investigated the impact of the cropping system (conventional and organic, with N availability expected to be lower in the latter one) on the antioxidant content of pigmented wheats. The authors observed that the cropping system significantly affected the contents of all the determined groups of antioxidant compounds, even though to a lesser extent than the year and the genotype, and this led to higher concentrations being observed in most of individual genotypes grown under the organic system. However, they only determined the total phenolic acid component. In the present study, the SPAs and CWBPAs showed opposite trends, which were reflected by the two most abundant identified phenolics, soluble sinapic acid and insoluble ferulic acid, thus indicating that the different forms of phenolic acid may respond differently to N fertilization rates as well as to various soil N contents over the compared environments. Further studies are therefore needed to investigate the biosynthesis mechanisms of different phenolic acid types, as there might be differences in the synthesis processes of soluble and insoluble fractions due to their specific storage sites, solubilities, and their roles within the plant organism. Furthermore, the individual genotypes reacted differently to increased soil N contents and increased N applications (data not shown) in terms of SPA and CWBPA contents, in a similar way to what Ma et al.^40^ observed in Pp wheat varieties supplied with different combinations of N and phosphorus fertilizer treatments, thus suggesting the importance of selecting specific genotypes that are naturally rich in bioactive compounds in suitable growing environments.

### Anthocyanins

Anthocyanins are a major class of red to blue flavonoid pigments that are extensively represented in plants but an unconventional and rare trait in wheat, where they differ significantly among genotypes and are highly correlated with the grain pigmentation. No pigmented forms of bread wheat existed in the past, but then breeders took advantage of and introgressed the ability to accumulate purple or blue pigments from tetraploid and diploid landraces or wild relatives of wheat.^3^ Black lines were later developed through the hybridization of Pp and Ba wheats.^5^ In the present study, the genotype factor had a significant impact on the total variance, on average accounting for 46% of the observed variation (Table 4). The total anthocyanin content (TAC) ranged between 1.2 mg·kg^–1^ of AF Jumiko (Pp) and 42.9 mg·kg^–1^ of AF Zora (Pp + Ba), as shown in Table 5. No anthocyanins were detected in the control variety or in the Ye genotypes, in either wheat or tritordeum. Other studies have stated that the grain color and anthocyanin content are mainly controlled by the genotype.^21,26^ Considerable differences in the total contents were also observed among the individual color groups (Figure 2E). The highest TAC was observed in the Pp + Ba genotypes (34.4 mg·kg^–1^), whose whole-meal flour was on average characterized by 5.1 and 1.7 times greater contents than those of the Pp (6.8 mg·kg^–1^) and Ba (19.9 mg·kg^–1^) wheats. Anthocyanins have been identified and quantified in other studies in various pigmented-grain wheats, with different contents and compositions. Ficco et al.,^21^ by means of HPLC analysis, observed total mean contents of 11.8 and 68.4 mg·kg^–1^ for Pp durum wheat and Ba bread wheat genotypes, respectively, while the total content of anthocyanins observed by Hosseinian et al.^41^ in Pp wheat samples was 447.6 mg·kg^–1^. These differences between studies could be attributed to the variations in wheat genetics and physiology (which depend on the donor and recipient cultivars used for the development of the germplasm), the environmental conditions, the extraction and quantification methods, and to the number of identified compounds.^3^ Overall, the results of the present study are in agreement with earlier works in which Pp + Ba genotypes were characterized by the highest TAC and were followed by Ba and Pp lines.^27,35,42^ The analysis of individual anthocyanins showed that the genotypes grouped according to the grain color not only differed concerning their total content but also in the anthocyanin profile (Figure 2E). As in previous studies, the most common anthocyanin in Pp wheats was found to be cyd-3-glu, followed by pnd-3-glu.^27,42−44^ Abdel-Aal et al.^43^ and Escribano-Bailón et al.^44^ found dpd-3-glu to be the most abundant anthocyanin in Ba wheats, while Sharma et al.^27^ found cyd-3-rut made a higher contribution. In this study, dpd-3-rut accounted for 45 and 38% of the total anthocyanins in the Ba and Pp + Ba groups, respectively, while dpd-3-glu and cyd-3-rut were the second most abundant ones. Delphinidin glycosides, which are responsible for the blue color, were mainly detected in the Ba and Pp + Ba groups, while cyanidin and peonidin glycosides were detected above all in the Pp group, similarly to what Giordano et al.^45^ reported. Furthermore, the Pp + Ba wheats exhibited a larger proportion of the glycosides mainly detected in the Pp genotypes than the Ba wheats.

In addition to the genotypic component, growing conditions can also affect anthocyanin accumulation.^41^ As shown in Table 4, the environment factor accounted for around 49% of the observed variation, followed by the G × E effect (4%), which was the only significant interaction observed for TAC. The smallest concentration of anthocyanins was observed in A (Table 5), and increasing values were observed in the B (1.4 times) and C (2.3 times) environments (9.8 vs 13.4 and 22.9 mg·kg^–1^). This trend was observed in all the individual genotypes at different levels, with the exception of AF Zora (Pp + Ba), which on average showed the highest TAC for the plots in the B environment (data not shown). The effect of different N treatments was limited (0.6%), although still significant, and consistent with the effect of the environment, according to the soil N content, with higher values recorded for 0 and 80 kg of N·ha^–1^ than for the 160 supply (+9 and +6%). In agreement with the findings of the present study, Fan et al.^26^ observed an increased accumulation of anthocyanins in Ba, Pp, and bread wheat grains for reduced applications of Zrcková et al.^39^ reported a higher TAC in the organic cropping system and assumed that the probable N deficiency of organically cultivated plants led to a higher surface/volume ratio of the wheat kernels and, thus, to a higher percentage of the pericarp and of the aleurone layer, where the majority of antioxidant compounds, including anthocyanins, accumulate. Our data related to TKW do not support this hypothesis, as the increased soil N content in the present work led to a higher tiller capacity and, thus, to smaller kernels. The observed correlations between TKW and the analyzed bioactive compounds were dependent on the investigated genotypes (data not shown).

The greater anthocyanin accumulation observed for low N levels might be ascribable to the stimulation of the phenylpropanoid pathway as a result of the N limitation, as previously mentioned for phenolic acids, and it might explain the much greater variation observed between environments compared to the different N fertilization rates, where differences in N limitation could probably be less marked, as reported by the plant agronomic response. Furthermore, anthocyanin-specific genes, as well as the genes involved in anthocyanin glycosylation and sequestration in the vacuole, were found to be highly expressed in response to nutrient deficiency and downregulated under a high N fertilizer application.^26,46^ However, the majority of findings about anthocyanin response to nutrient signaling at a genetic and molecular level are based on *Arabidopsis* and fruit crops.^38,46^ The increased interest in pigmented cereals registered globally will require the transfer of knowledge to cereal crops.

### Carotenoids

Carotenoids are characterized by a wide variety of biological functions, in both plant and human tissues, and these functions are mainly determined by their molecular structure.^4^ Their profile in cereals is mainly composed of xanthophylls (the oxygen-containing carotenoids), and lutein is usually the most abundant in wheat grains, followed by zeaxanthin, antheraxanthin, and small amounts of carotenes (hydrocarbons that contain no oxygen), that is, α- and β-carotenes.^3^ Carotenoids predominantly occur in plants as *E*-isomers, but *Z*-isomers of lutein and zeaxanthin can be detected, as a consequence of the photochemical isomerization of their all-*E*-isomers, which involves changes in their biological properties, bioavailability, and in their antioxidant activity.^47^ Furthermore, some cereals have the ability to form xanthophyll mono- and diesters, which enables the accumulation of greater amounts of pigments in grains and, according to the findings of some authors, protects xanthophylls against degradation and improves bioaccessibility during digestion.^48^ Lutein esterified with fatty acids has not been found in bread or durum wheat grains, or it has been found at low concentrations, while a much larger degree of esterification has been observed in tritordeum cultivars.^6^

Three-way ANOVA (Table 4) showed significant effects of all three factors on the total carotenoid content (TCC), although genotype accounted for most of the observed variation (>97%). This is in agreement with the high heritability and the low G × E interaction that characterizes this trait, which has led to a wide variation in the content of carotenoids among cereal species and varieties.^48^ As shown in Figure 2F, the anthocyanin-rich varieties were found to have a low TCC, with the lowest concentration observed in the Pp + Ba group (1.5 mg·kg^–1^). The Ba and Pp genotypes did not differ significantly from the red-grained control, while the Ye wheat had the highest TCC among the pigmented wheats (5.6 mg·kg^–1^). Overall, tritordeum presented the highest TCC (8.8 mg·kg^–1^), which was 5.8, 5.0, 4.6, 4.3, and 1.6 times higher than the Pp + Ba, Ba, red, Pp, and Ye wheats. Differences were also found within the same color group (Table 5), especially among wheats with Pp, due to the wide multiplicity of evaluated genotypes, with a marked superiority of the AnthoGrain^TM^ genotype (2.7 mg·kg^–1^), followed by Rosso and KM 106-18 (2.6 and 2.1 mg·kg^–1^). The carotenoid that was detected the most was lutein, which represented 66% of the total content (a 2.64 mg·kg^–1^ average of all the genotypes). Lutein esters and zeaxanthin were also found in smaller amounts, accounting for 15 and 14% of the total content, while lutein *Z*-isomers on average represented <5%. The carotenoid profiles were similar in the red, Pp, Ba, and Pp + Ba wheats, while the average zeaxanthin content of the Ye genotypes differed and was only 6% of the total carotenoids, compared to the other color groups (average of 19%). Furthermore, the Ye wheat was characterized by a higher share of lutein esters than all the other analyzed genotypes (34 vs 10%) and by the highest absolute value (1.89 mg·kg^–1^). Lutein *Z*-isomers were mainly found in tritordeum (0.63 vs 0.09 mg·kg^–1^).

Despite the high genetic effect on carotenoid accumulation, growing conditions have also been reported to cause changes in TCC and individual compounds.^10,48^ In the present study, the environment had a significant effect on TCC, as did N fertilization, but, overall, they accounted for a small share of the total variation, that is, 1.2 and 0.1%, respectively (Table 4). The effects of the G × E, G × N, and E × N interactions were very limited (1.0, 0.1, and 0.1%, respectively), although still significant (Table 4). The findings of previous studies investigating the effect of N fertilization on the carotenoid content are contradictory, with an overall limited role for this parameter and a much more variation being attributed to differences in weather conditions between years, mainly in the average temperature patterns and rainfall distribution.^10,15,48^

### Antioxidant Capacity of the Whole-Meal Flour and Multivariate Analysis

The obtained results showed a highly significant effect of the genotype on AC, which was determined by employing ABTS and FRAP methods (2 and 29% of the observed variation; Table 4). Tritordeum showed the highest AC_ABTS_ (18.5 mmol of TE·kg^–1^), followed by the Ye wheat (16.8 mmol of TE·kg^–1^), while their AC_FRAP_ was significantly lower (10.2 mmol of TE·kg^–1^, mean value of the tritordeum and Ye wheat) than the Pp + Ba and Pp groups (mean value of 11.8 and 11.2 mmol of TE·kg^–1^), and did not differ from that of the red and Ba genotypes (Figure 2G,H). The Pp + Ba group recorded the highest AC_FRAP_, and the two black genotypes did not differ significantly from each other (Table 5). A wide variability was observed within the Pp group, with AnthoGrain^TM^ presenting the highest measured AC for both assays (17.4 mmol of TE·kg^–1^ of AC_ABTS_ and 12.2 mmol of TE·kg^–1^ of AC_FRAP_). Overall, the results obtained by the ABTS assay were more heterogenous among genotypes and higher than those obtained by FRAP. These two assays are widely used in the characterization of foods, and their reactivity toward antioxidants is very different: this may explain the significant difference between their reported patterns when sorted by pigmented groups (Figure 2G,H).

The environment was the most prominent factor contributing to the overall variation observed for both AC_ABTS_ and AC_FRAP_ (97 and 47%), which may be attributable to the greater effect of this factor on the individual groups of monitored analytes, except for the TCC (Table 4). Nevertheless, more marked differences were observed for AC_ABTS_ than AC_FRAP_ and the results differed, depending on the considered type of assay (Table 5), with AC_ABTS_ presenting higher levels in the C and B environments compared to A (+55 and +10%) and AC_FRAP_ being the highest in the A environment (+8% compared to B, which provided the lowest mean value). The effects of the interaction were limited (<1%) for AC_ABTS_, although still significant, while G × E was the most important interaction for AC_FRAP_, accounting for 12% of the observed variation (Table 4), due to different responses of the genotypes in the compared environments.

Phenolic compounds have been associated with the ability to capture reactive oxygen species in other studies on cereals, such as wheat,^21,45^ and it has been pointed out that an enhanced accumulation of biologically active compounds, such as anthocyanins and carotenoids, in pigmented cereal grains can lead to additional effects.^27^ In the present study, the procedure adopted for the determination of the AC with both of the assays did not involve sample treatments such as chemical or enzymatic hydrolysis prior to the measurement. As shown by Serpen et al.,^49^ the direct analysis of whole-meal samples enables to measure the total AC of cereals, allowing the soluble, but also the insoluble fraction with functional groups on the surface, to simultaneously come into contact with the ABTS radical and with the ferric ions of the FRAP assay, taking advantage of the surface reaction occurring at the solid–liquid interface. The direct procedure was applied in order to retain the synergistic effect of antioxidants that is partially lost when single compounds are extracted and analyzed for the AC^23^ and to better relate the data with the antioxidant effects exerted in the human gastrointestinal tract, where a simultaneous action of all the antioxidants present in the samples is expected.^1^ However, using different methods to estimate the scavenging or reducing activity of an extract containing solid matrix can lead to different results. The different response of AC_ABTS_ and AC_FRAP_ to the genotype and the environment factors can be explained by considering the chemical mechanisms and the solvent compositions of the assay solutions. Although the redox potential is comparable between the assays, the reaction conditions differ, particularly the pH at which the reaction is performed, and the steric requirements of the oxidizing molecules and the ferric *di*-TPTZ and ABTS.^50^ Moreover, the reaction medium significantly affects the measured AC, as it acts not only as a reactant carrier but also as a solubilizer of the food matrix.^24^ The ABTS aqueous solution was mixed with ethanol in a 1:1 (*v*/*v*) ratio in order to enhance the interaction of both hydrophilic and lipophilic antioxidants with the radicals, while the FRAP assay was performed in aqueous acetic buffer (pH 3.6) to maintain iron solubility. Thus, the ABTS radical could reach extensive range of compound polarities and react with a greater amount of compounds than the ferric ions of the FRAP assay.^24^ Due to their polarity, the SPAs are highly soluble in water–alcohol solutions, such as ethanol or methanol, and are therefore highly soluble in the ABTS reaction medium. At the same time, xanthophylls exhibit low polarity and they are more likely to go into the aqueous ethanolic solution of the ABTS radical, while their solubility will be negligible in the aqueous solution of the FRAP assay.^23^ In addition, the acidic environment of the FRAP system could negatively affect the stability of carotenoids.^51^ On the other hand, the highly hydrophilic anthocyanins are more likely to affect the AC determined by the FRAP method. The water solvent in the FRAP system may also contribute to the opening of starch, protein, and cellulose structures that act as physical barriers to liquid diffusion and tend to shrink in ethanol environment, thereby enhancing the interactions at the solid–liquid interface between the antioxidant groups bound to the insoluble polysaccharide fraction and the ferric ions.^24^ Finally, it should also be considered the different reactivity of the two assays toward other antioxidant components of the matrix not measured in the present study, such as thiol-type antioxidants, which are not effectively oxidized within the FRAP protocol time.^52^

**Figure 3:**
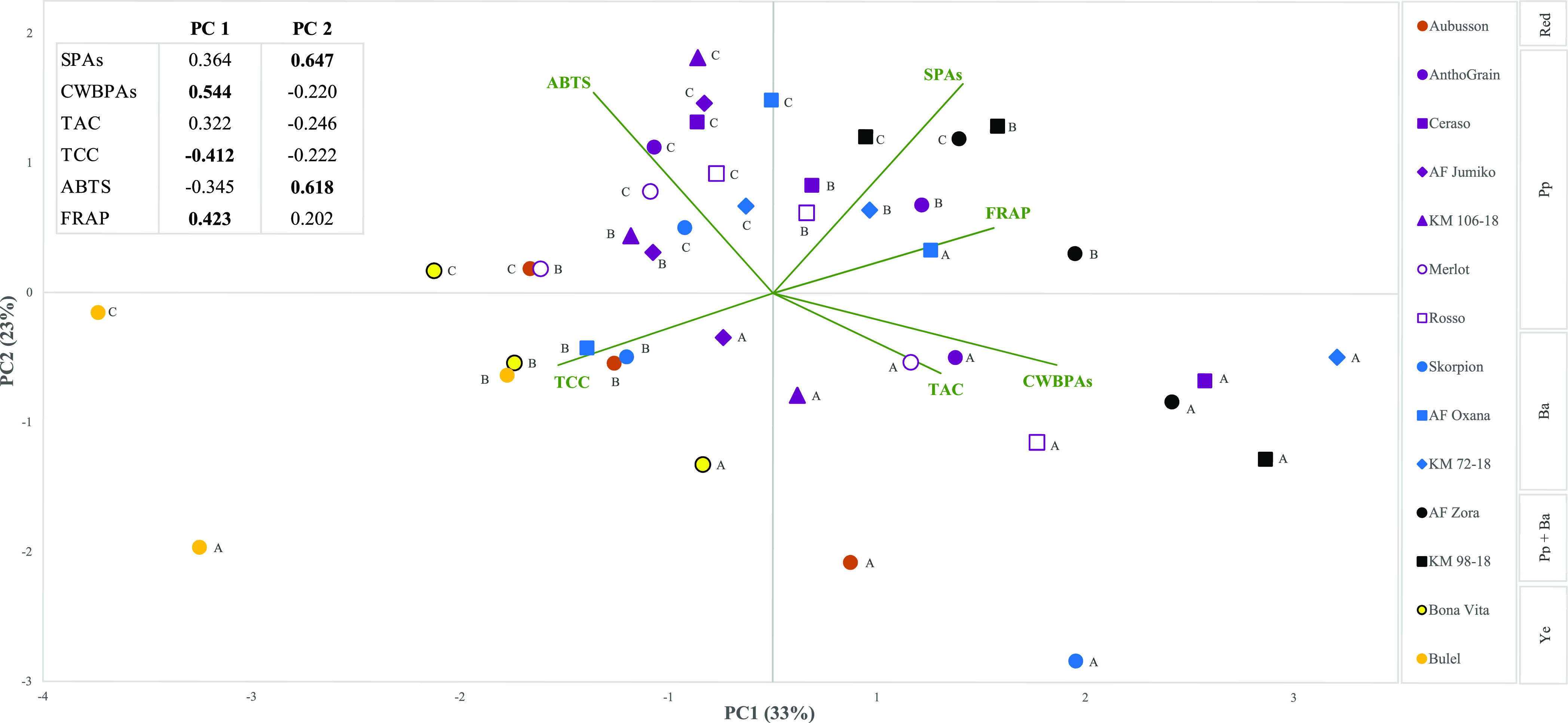
PCA biplot of the analyzed genotypes (*n* = 378) and phytochemical parameters associated with health-related substances. The table on the left-hand side shows the loadings of the variables for the first two principal components. Loadings greater than 0.4 are indicated in bold. The length of each variable vector is proportional to its contribution to separating the genotypes, and the direction of the vectors indicates their relative contribution to PC1 and PC2. The sample plots are grouped by genotype and by environment, on the basis of the average mean of 3 N levels and 3 replications. The color names on the right-hand side indicate the color of the kernels. Pp, purple pericarp; Ba, blue aleurone; Pp + Ba, black grain; Ye, yellow endosperm. Environments: A (Cigliano, 2019–20), B (Cigliano, 2018–19), C (Carmagnola, 2018–19). SPAs, soluble phenolic acids; CWBPAs, cell wall-bound phenolic acids; TAC, total anthocyanin content; TCC, total carotenoid content; ABTS, antioxidant capacity determined by means of ABTS assay; FRAP, antioxidant capacity determined by means of FRAP assay.

In view of these differences, multivariate analyses were carried out to quantitatively analyze and better understand the relationships between the analyzed health-related compounds and the antioxidant properties of the genotypes studied under different growing conditions. A PCA was performed on the 14 analyzed genotypes (*n* = 378), and a biplot was produced, where the two first principal components explained 33 and 23% of the total variation of the samples (Figure 3). Principal component 1 (PC1) mainly appeared to differentiate the entries according to their CWBPAs and TCC and to their AC_FRAP_, whereas the SPAs and AC_ABTS_ were the most important contributors to principal component 2 (PC2). Furthermore, variables with longer vectors (CWBPAs, SPAs, and AC_ABTS_) were more discriminating of the entries than shorter ones. The biplot showed a characteristic clustering of data, as TCC, SPAs, and AC_FRAP_ contributed more to separating the samples according to the genotype, while TAC, CWBPAs, and AC_ABTS_ differentiated the samples according to the environment. Indeed, samples from the C site were all placed on the lower right side of the biplot, as a result of their high concentrations of CWBPAs and anthocyanins, while the B and A plots were placed in the upper part, close to AC_ABTS_ and the SPAs. When the data are sufficiently approximated by the biplot, the cosine of the angle between the vectors of two variables approximates the correlation coefficients between them. The loading plots showed a positive correlation between the CWBPAs, TAC, and AC_FRAP_ and a negative relationship of the latter ones with TCC and AC_ABTS_. The SPAs were the only phytochemicals to be positively correlated with the AC assessed with both methods, highlighting the different contribution of various bioactive compounds to the AC estimated by different assays, even when performed on solid samples.

In short, the wide array of genotypes with different genetic backgrounds analyzed in the present study provides a deeper understanding of the response of differently pigmented genotypes to environmental conditions and N fertilization rates, regarding agronomic and qualitative traits and phytochemical accumulation. Overall, the antioxidant profiles resulted to be strongly influenced by the genotype. The environment played a significant role, which could be partly ascribed to the soil N content, according to the conditions compared and the agronomic effects reported. The N fertilization treatments affected antioxidant accumulation similarly but to a lesser extent than the environment. Larger concentrations of SPAs were observed in the plots grown in soil characterized by a high N content, whereas the CWBPAs and anthocyanins were significantly reduced by a high soil N content and higher N fertilization rates. The greatest differences in the bioactive compound content were detected among the color groups, with the Pp + Ba group accumulating the highest content of anthocyanins and phenolic acids and the Ye group being the richest in carotenoids. However, a wide variability was also observed among the cultivars belonging to the same color group, and some of them appear to be promising for the development of innovative supply chains and the production of health-valued foods, as they have shown good yield and quality performances, and retained good antioxidant features in all the considered production systems. Therefore, a genetic improvement and the selection in specific environments are of key importance to develop pigmented genotypes characterized by high phytochemical accumulation and satisfactory yield potential, when compared to commercially diffused varieties. N fertilization affected the agronomic and qualitative traits, but overall had limited effects on some bioactive compounds. Thus, an optimized N management can contribute to the efficient growth of these phytonutrient-rich varieties, as it plays a fundamental role in enhancing the yield capacity and in ensuring a good technological quality and desirable product-making features, according to the intended use of the grains, without compromising their high value in terms of phytochemicals. Further studies will be necessary to improve the knowledge related to disease resistance and safety aspects of pigmented genotypes, as well as to post-harvest processing, to generate the interest of growers and producers, and to favor the large-scale adoption of such varieties.

## Supporting Information Available

Appearance of the ears of the wheat and tritordeum genotypes compared in the study (Figure S1), timing of the main agronomic management practices applied to all the plots in the field experiments (Table S1), effect of the genotype, the environment, the N fertilization, and their interaction on the qualitative parameters of whole-meal flour (Table S2), and technological parameters, farinographic assessment, and baking test of wheat samples from the field experiments conducted in the 2018–19 and 2019–20 periods at Cigliano, Italy, with 80 kg of N·ha^–1^ (Table S3) (PDF)

## Abbreviations

ABTS: 2,2′-azino-bis(3-ethylbeenzothiazoline-6-sulfonic acid) diammonium salt
AC: antioxidant capacity
ANOVA: analysis of variance
AUCGC: area under the canopy greenness curve
BHT: butylated hydroxytoluene
CWBPAs: cell wall-bound phenolic acids
cyd-3-glu: cyanidin-3-glucoside
cyd-3-rut: cyanidin-3-rutinoside
dpd-3-glu: delphinidin-3-glucoside
dpd-3-rut: delphinidin-3-rutinoside
DW: dry weight
E: environment
FRAP: ferric reducing antioxidant power
G: genotype
GDDs: growing degree days
GPC: grain protein content
GS: growth stage
GY: grain yield
HPLC/DAD: high-performance liquid chromatography with diode array detection
LOD: limit of detection
N: nitrogen
NDVI: normalized difference vegetation index
PCA: principal component analysis
pnd-3-glu: peonidin-3-glucoside
REGW-F test: Ryan–Einot–Gabriel–Welsch F test
SPAs: soluble phenolic acids
TAC: total anthocyanin content
TCC: total carotenoid content
TE: Trolox equivalents
TKW: thousand kernel weight
TPTZ: 2,4,6-tris(2-pyridyl)-*s*-triazine
TW: test weight
