# Supplementary material for: Influence of Agronomic Practices on the Antioxidant Compounds of Pigmented Wheat (Triticum aestivum spp. aestivum L.) and Tritordeum (× Tritordeum martinii A. Pujadas, nothosp. nov.) Genotypes
Source: J Agric Food Chem. 2023 Aug 29;71(36):13220–33. doi: 10.1021/acs.jafc.3c02592 (PMC10510394; doi:10.1021/acs.jafc.3c02592)
Supplement: Supplementary file 1 — jf3c02592_si_001.pdf [file jf3c02592_si_001.pdf]

JOURNAL OF AGRICULTURAL AND FOOD CHEMISTRY

SUPPORTING INFORMATION AVAILABLE FOR PUBLICATION

Influence of agronomic practices on the antioxidant compounds of pigmented wheat (*Triticum aestivum* spp. *aestivum* L.) and tritordeum (× *Tritordeum martinii* A. Pujadas, nothosp. nov.) genotypes

Authors:

Claudia Sardella[a], Barbora Burešová[b], Zora Kotíková[b], Petr Martinek[c], Raffaele Meloni[a], Luboš Paznocht[b], Francesca Vanara[a], Massimo Blandino[a*]

Affiliation:

[a]Department of Agricultural, Forest and Food Sciences, University of Turin, Largo Paolo Braccini 2, 10095 Grugliasco, Turin, Italy.

[b]Department of Chemistry, Faculty of Agrobiology, Food and Natural Resources, Czech University of Life Sciences Prague, Kamýcká 129, 165 00 Prague-Suchdol, Czech Republic.

[c]Agrotest Fyto, Ltd., Havlíčkova 2787/121, 767 01, Kroměříž, Czech Republic.

*Corresponding author: Massimo Blandino.

Phone: +39 0116708895. E-mail: massimo.blandino@unito.it.

Figure S1. Appearance of the ears of the wheat and tritordeum genotypes compared in the study.

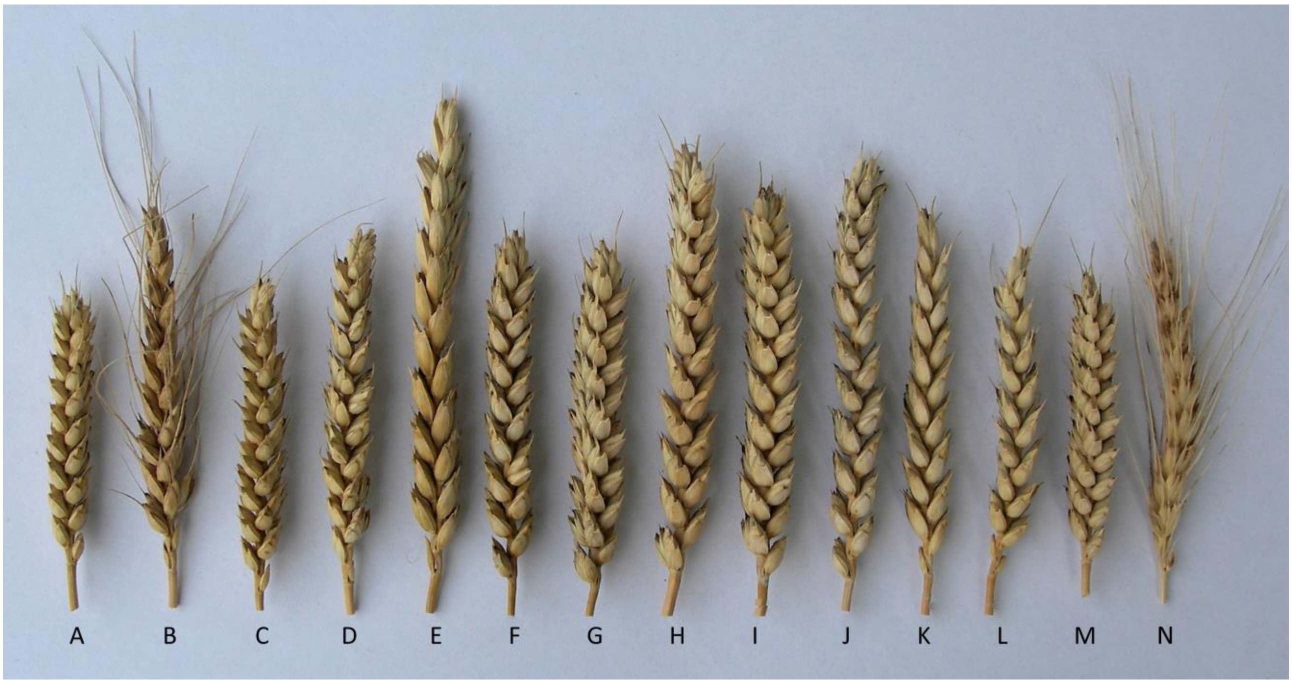

A-M – *Triticum aestivum* spp. *aestivum* L.: A, Aubusson; B, AnthoGrain™; C, Ceraso; D, AF Jumiko; E, KM 106-18 – this material has a long glume transferred from *Triticum polonicum* L.; F, Merlot; G, Rosso; H, Skorpion; I, AF Oxana; J, KM 72-18; K, AF Zora; L, KM 98-18; M, Bona Vita.

N – × *Tritordeum martinii* A. Pujadas, nothosp. nov.: Bulel.

Table S1. Timing of the main agronomic management practices applied to all the plots in the field experiments conducted in the 2018-20 period in north-west Italy.

**Crop technique**	**Growth stage**	**A**<br>Cigliano (VC), 2019-20	**B**<br>Cigliano (VC), 2018-19	**C**<br>Carmagnola (TO), 2018-19
Sowing date		6 Nov. 2019	16 Nov. 2018	14 Nov. 2018
N fertilization	Tillering (GS 23)	5 March 2020	8 March 2019	4 March 2019
	Stem elongation (GS 32)	3 April 2020	8 April 2019	9 April 2019
Fungicide + insecticide	Anthesis (GS 65)	6 May 2020	16 May 2019	22 May 2019
Harvest date		30 June 2020	2 July 2019	10 July 2019

Table S2. Effect of the genotype, the environment, the N fertilization, and their interaction on the qualitative parameters of the whole-meal flour of the investigated wheat and tritordeum genotypes.

**Factor**	**Source of variation**		**TW ($kg \cdot hL^{-1}$)**		**GPC (%)**		**Ash (%)**	
**Genotype (G)**	Red	Aubusson	78.0	de	11.5	def	1.81	efg
	Pp	AnthoGrain™	78.9	c	11.9	bcde	1.92	a
		Ceraso	78.2	cd	11.4	de	1.78	g
		AF Jumiko	81.1	a	11.3	e	1.83	cde
		KM 106-18	76.9	f	11.8	bcdef	1.90	a
		Merlot	78.7	c	11.6	cdef	1.80	fg
		Rosso	79.6	b	11.4	de	1.86	bc
	Ba	Skorpion	72.9	i	13.2	a	1.87	b
		AF Oxana	74.8	g	12.1	bcd	1.82	def
		KM 72-18	73.9	h	12.1	bc	1.84	bcde
	Pp + Ba	AF Zora	77.5	e	12.3	b	1.90	a
		KM 98-18	78.7	cd	12.3	bc	1.86	bc
	Ye	Bona Vita	79.6	b	12.9	a	1.85	bcd
	Trit	Bulel	74.6	g	12.9	a	1.90	a
		p (F)	***		***		***	
**Environment (E)**		A	76.8	c	11.1	c	1.83	c
		B	78.1	a	11.3	b	1.84	b
		C	77.2	b	13.8	a	1.89	a
		p (F)	***		***		***	
**N Fertilization (N) (kg of $N \cdot ha^{-1}$)**		0	77.7	a	11.5	c	1.85	b
		80	77.3	b	11.8	b	1.84	b
		160	77.2	b	12.9	a	1.87	a
		p (F)	***		***		***	
**G × E**	*p* (F)		***		***		***	
**G × N**	*p* (F)		*		ns		ns	
**E × N**	*p* (F)		*		***		ns	
**G × E × N**	*p* (F)		**		ns		ns	

Pp, purple pericarp; Ba, blue aleurone; Pp + Ba, purple pericarp + blue aleurone (black); Ye, yellow endosperm; Trit, tritordeum; TW, test weight; GPC, grain protein content. The results are expressed on a DW basis. Means followed by different letters are significantly different, according to the REGW-F test [(*) p (F) < 0.05, (**) p (F) < 0.01, (***) p (F) < 0.001, and ns, non-significant].

Table S3. Technological parameters, farinographic assessment, and baking test of the refined flours of wheat samples from the field experiments conducted in the 2018-19 and 2019-20 periods at Cigliano, Italy, supplied with 80 kg of N·ha^{-1}.

		**Technological parameters**				**Farinographic assessment**						**Baking test**				**Bread appearance**	
**Factor**	**Source of variation**	**HFN (s)**	**ZT (mL)**	**GI**	**WGC (%)**	**WA (%)**	**DT (min)**	**DS (min)**	**DS$_{(10)}$ (FU)**	**DS$_{(12)}$ (FU)**	**FQN**	**LV (cm^3)**	**LH (mm)**	**LW (mm)**	**SPV (ml·100 g^{-1})**	**LS**	**CC**
**Genotype**	Aubusson	333	18.3	96.7	17.6	52.3	1.3	1.5	104	114	20.5	283	51	95	285	arched	normal
	AnthoGrain™	294	16.7	93.4	20.5	56.8	1.4	1.7	81	97	25.5	243	51	86	233	arched	normal
	Ceraso	217	26.5	94.5	22.9	60.3	1.5	1.0	137	162	21.0	283	55	97	268	arched	dark
	AF Jumiko	350	17.3	95.0	21.5	59.2	1.4	1.4	105	125	23.8	240	49	89	229	arched	light
	KM 106-18	226	12.0	84.5	23.8	53.8	1.2	1.3	130	151	19.5	270	45	100	266	arched	normal
	Merlot	254	26.3	97.3	19.6	62.2	1.5	1.3	104	121	25.5	273	51	92	255	arched	light
	Rosso	228	19.7	98.7	15.6	60.4	1.6	1.8	81	98	29.8	247	49	93	241	arched	normal
	Skorpion	160	29.3	85.3	28.0	59.7	1.6	1.4	127	153	25.0	263	51	102	252	arched	dark
	AF Oxana	301	30.3	96.0	25.5	60.5	1.7	1.3	119	141	25.3	263	46	98	250	arched	normal
	KM 72-18	102	27.0	68.2	28.1	59.3	1.6	1.2	143	169	23.0	300	30	134	321	flattened	normal
	AF Zora	230	21.7	65.1	28.4	58.8	1.7	1.4	115	137	25.8	257	50	101	254	arched	normal
	KM 98-18	186	24.3	89.2	24.5	61.0	1.4	1.0	135	158	21.5	297	48	99	307	arched	normal
	Bona Vita	239	35.5	95.9	26.2	58.3	1.7	1.7	87	105	26.5	270	56	94	254	arched	normal
**Harvest year**	2019	243	22.6	90.7	21.8	55.3	1.5	1.2	116	139	23.0	272	47	101	262		
	2020	237	24.3	87.7	24.7	62.0	1.5	1.6	110	127	25.1	265	50	96	263		

HFN, Hagberg falling number; ZT, Zeleny test; GI, gluten index; WGC, wet gluten content; WA, water absorption; DT, development time; DS, dough stability; DS$_{(10)}$, degrees of softening after 10 min; DS$_{(12)}$, degrees of softening 12 min after the maximum; FQN, farinographic quality number; LV, loaf volume; LH, loaf height; LW, loaf width; SPV, specific pastry volume; LS, loaf shape; CC, crust color; FU, farinographic unit.

HFN was determined according to ISO 3093:2009, using a Perten LM 3120 mill and 7 g of flour on a 15% moisture basis. ZT was determined according to ISO 5529:2007, using a Brabender Sedimat mill. GI and WGC were determined according to ICC Standard No. 155, using a Perten Glutomatic 2200 and Centrifuge 2015. The rheological properties were determined by means of a farinograph (ICC Standard No. 115/1), producing dough with a consistency of 500 FU. A baking test was performed using the authors' own method. The recipe is based on ICC Standard No. 131. The other components in the bread formula were instant yeast (1.8% of the total weight), salt (1.5%), sugar (1.86%), ascorbic acid (0.005%) and water. Flours with a higher falling number than 250 s were supplemented with malt flour. The dough was prepared in a farinograph with a 300 g mixer.
